# Bilateral gluteal metastases from a misdiagnosed intrapelvic gastrointestinal stromal tumor

**DOI:** 10.1186/1477-7819-6-139

**Published:** 2008-12-30

**Authors:** Dritan Pasku, Apostolos Karantanas, Elpida Giannikaki, Maria Tzardi, Emmanouil Velivassakis, Pavlos Katonis

**Affiliations:** 1 Department of Orthopaedic and Traumatology, University Hospital of Heraklion, Crete, Greece; 2 Department of Radiology, University Hospital of Heraklion, Crete, Greece; 3 Department of Pathology, University Hospital of Heraklion, Crete, Greece

## Abstract

**Background:**

The location of gastrointestinal stromal tumors (GIST) outside of the gastrointestinal system is a rare event.

**Case presentation:**

A 56-year old woman presented with a GIST of the pelvis was misdiagnosed and treated as a uterine leiomyosarcoma. The diagnosis was made after the CD117 (KIT) positivity in the biopsy of the excised bowel mass four years from the first presentation. During this period she presented a bilateral muscle and subcutaneous metastasis in the gluteal area.

**Conclusion:**

The correct diagnosis of the extra-gastrointestinal stromal tumor is a challenge even for experienced pathologists. CD117 (KIT) positivity is the most important immunohistochemical feature in the histological diagnosis. To our knowledge a metastatic EGIST (extra-gastrointestinal stromal tumor) to the skeletal muscle bilaterally has not been described previously in the English medical literature.

## Background

Gastrointestinal stromal tumors (GISTs) are the most frequent mesenchymal neoplasms that may occur in any segment of the gastrointenstinal tract. In the earlier medical literature GISTs were diagnosed as smooth muscle tumors (leiomyoma, leiomyosarcoma and leiomyoblastoma) or tumors of the peripheral nerves (neurofibroma and schwannoma). Nevertheless, the application of immunochemistry has reclassified them. GISTs are mostly found in the stomach (60–70%) and in the small intestine (20–30%). The location in the esophagus and in the colon is about 5% [1,2]. Extra-gastrointestinal stromal tumors (EGISTs) may occur in the mesentery, omentum and retroperitoneun in about 5% of all cases [3,4].

The GISTs are usually positive for CD117 (KIT), which is the specific defining immunohistochemical feature for this group of tumors. The accurate surgical resection and the postoperative therapy with the single-agent KIT inhibitor imatinib mesilate (Gleevec, Glivec) is the gold standard treatment for GISTs. Post-operative recurrence is observed in 40–90% of the cases treated with surgery alone [5].

The differential diagnosis between GISTs and other similar tumors is a challenge. GISTs usually metastasize to the liver and lung, but in recent medical literature an intracranial metastasis from a perisacral GIST has been reported [6]. To the best of our knowledge, metastasis to skeletal muscles and subcutaneous fat has never been described for EGISTs. We present herein, the natural history and gluteal bilateral soft tissue secondary deposits from a misdiagnosed and treated only surgically intrapelvic GIST.

## Case presentation

A 56-year old woman was presented to the orthopaedic department with a 3-month history of a painless mass in the upper external area of the left gluteus.

**Figure 1:**
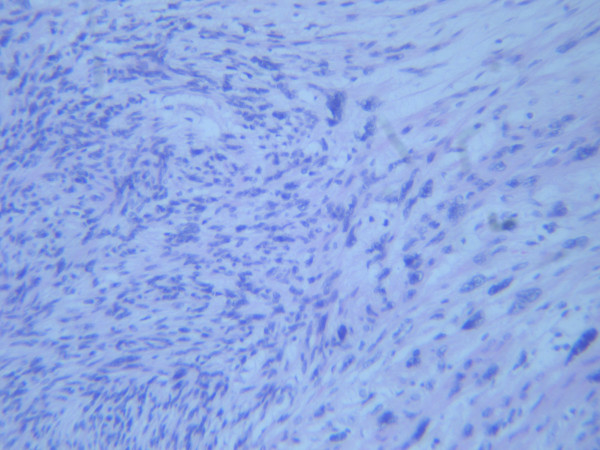
**Microscopic photogragh of the first tumor, showing interlacing bundles of spindle cells with medium atypia (×200, H&E)**.

**Figure 2:**
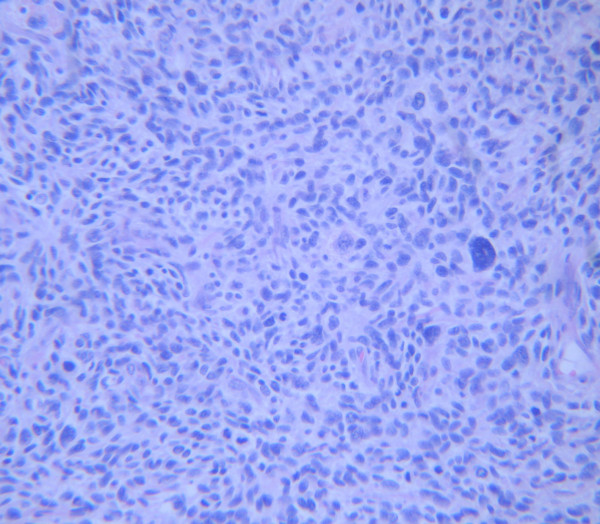
**Microscopic photograph of the first tumor showing giant cells and abundant mitosis (×200, H&E)**.

Two years, previously she underwent an abdominal hysterectomy because of an enlarging mass found in the pelvis, located between the uterus and the recto-sigmoid area. CA 125 was moderately elevated at 61.9 U/ml. The resected mass was bilobular (9 × 7 × 6, 5 cm and 5, 2 × 5 × 5 cm) with hard and partially soft elements. Multiple intramural uterine leiomyomas were also identified. The tumor was diagnosed as "a leiomyosarcoma" composed of cellular bundles of spindle cells with medium grade atypia and presence of giant cells. The range of mitosis was very high, up to 50/10 H.P.F (high power field). Immunohistochemical analysis with avidin-biotin-peroxidase complex showed positive reaction for vimentin, smooth muscle actin and desmin (Fig 1 and 2). The patient was treated post-operatively with radiotherapy and chemotherapy (Gemcitabine and docetaxel).

**Figure 3:**
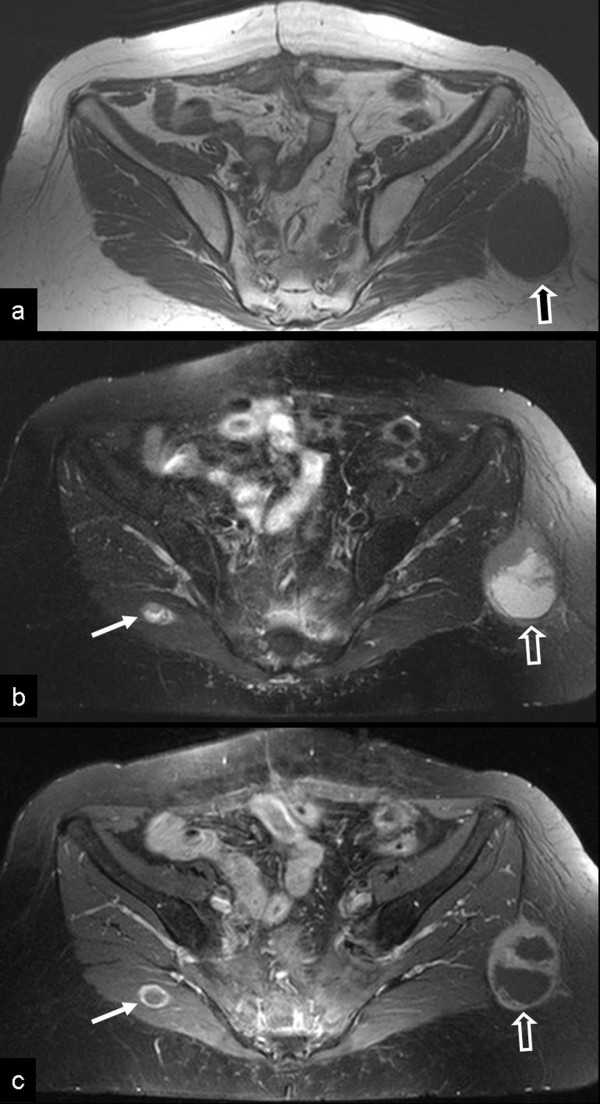
**Metastatic disease from extragastrointestinal GIST**. a) The axial T1-w MR image shows an intermediate signal intensity ovoid-shaped mass, lateral to the gluteus maximus muscle, within the subcutaneous fat (arrow). An intact fat plane separated the mass from the muscle. b) The fat suppressed T2-w TSE MR image, shows the high signal intensity of the central mass and the intermediate signal intensity of the anterior wall (open arrow). A second smaller lesion with similar imaging characteristics is shown in the right gluteus maximus muscle (thin arrow). c) The contrast enhanced fat suppressed T1-w SE MR image shows the peripheral enhancement of the wall (open arrow). The central non enhancing component presumably corresponds to necrosis. Ring-like enhancement is also shown in the smaller lesion (thin arrow).

One year after the hysterectomy, a single metastasis was diagnosed during a routine follow up in the left upper lung lobe treated surgically with lobectomy. The histological diagnosis was compatible with the known primary tumor. One year later, the patient was presented in the orthopaedic department with a painless mass in the left gluteus area. The clinical examination revealed a focal lump in the left gluteal area. The subsequent magnetic resonance imaging study, showed a mass with central necrosis located in the subcutaneous fatty tissue, in close proximity to the gluteal muscles. In addition, a second small lesion with similar findings was detected in the right gluteal muscle (Fig 3).

**Figure 4:**
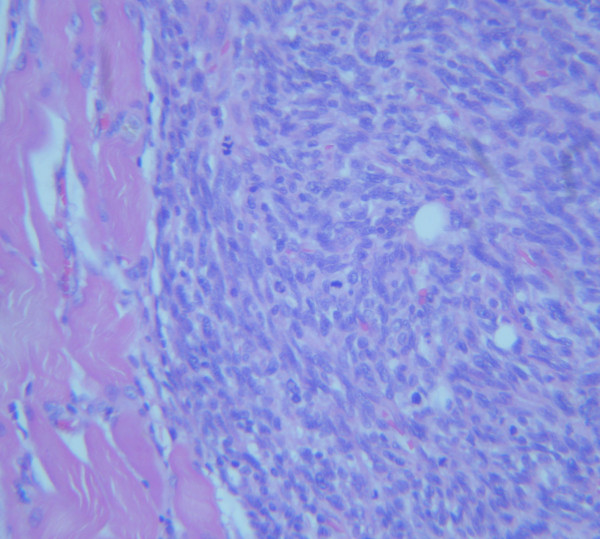
**Microscopic photograph, showing a metastatic lesion in the muscle (×200, H&E)**.

A diagnosis of soft tissue metastasic disease was suggested and a surgical resection of both masses was undertaken. The post-operative period was uneventful. The histological diagnosis confirmed the presence of a spindle cell sarcoma with central necrosis and morphologic features similar to the patient's previous sarcoma (Fig 4). A routine abdominal CT-Scan showed changes of the previous hysterectomy and an edematous appearance of the sigmoid colon wall, as well as presacral fat edema attributed to previous radiotherapy.

**Figure 5:**
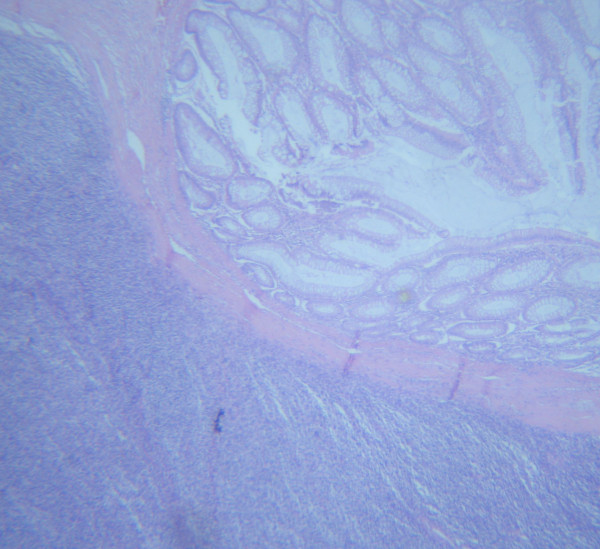
**Microscopic photograph, showing the tumor infiltrating the large bowel wall (×100, H&E)**.

**Figure 6:**
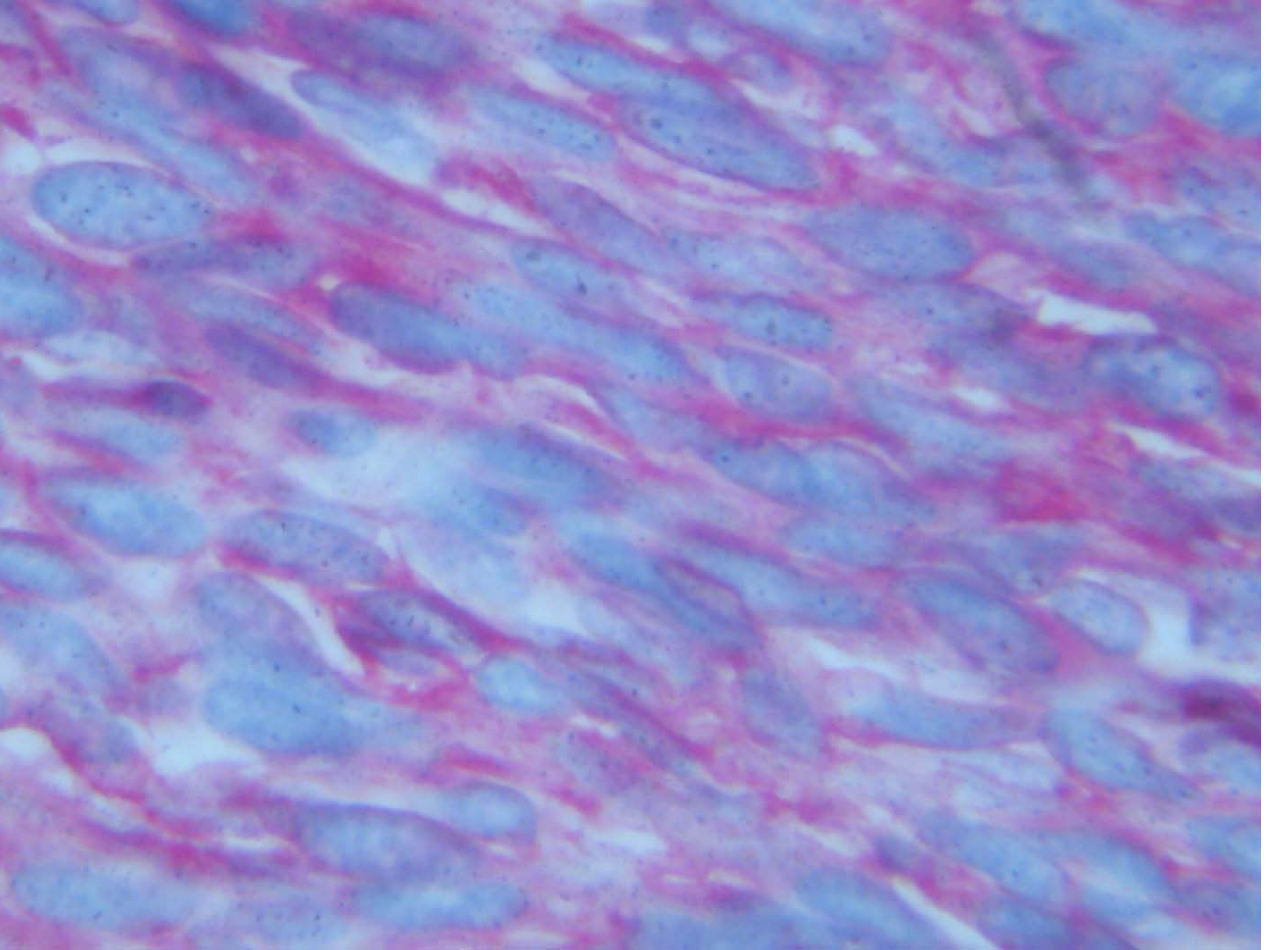
**Immunohistochemical stain for CD 117, showing positivity of the tumor cells in the large bowel (×400)**.

Four years after the hysterectomy the patient was presented at the emergency department with symptoms of intestinal obstruction. During laparotomy, a mass of 10.5 × 4.5 × 4 cm was identified infiltrating the bowel wall and protruding into the sigmoid lumen. A colectomy combined with colostomy was performed. Microscopically, the resected mass composed of interlacing bundles of spindle and epithelioid mesenchymal cells with morphological features similar to the previously described tumors. The immunohistochemical analysis of the cells showed positive for vimentin and a focal positivity for actin (α-SMA). This time, a CD117(c-KIT) immunohistochemical stain was performed and the neoplastic cells showed extensive positivity. A diagnosis of a mesenchymal stromal tumor was established (Fig 5 and 6). A retrospective analysis of the original tumor performing immunhistochemistry, showed a focal positivity of the neoplastic cells for CD117(c-kit) (Fig 5)

Based on these features, the primary pelvic tumor was most probably an EGIST originally misdiagnosed as leiomyosarcoma. Post-operatively the patient underwent therapy with Imatinib Mesylate (Gleevec, Novartis) and one year after surgery is now free of symptoms.

## Discussion

GISTs are mesenchymal tumors of the gastrointestinal tract, believed to originate from interstitial cells of Cajal or related stem cells. Cajal cells are intermediates between the gastrointestinal (GI) autonomic nervous system and the smooth muscle cells regulating GI motility and autonomic nerve function [7,8]. Gastrointenstinal mesenchymal tumors (GIMTs) are classified below: a) myogenic tumors that differentiate into smooth muscle cells such as leiomyoma (LM) and leimyosarcoma (LMS), b) neurogenic tumors that differentiate into neurocytes such as schwannoma and c) GIST that differentiate into other cell types. In general, about 80% of GIMT are GIST, 15% are myogenic tumors and 5% are neurogenic tumors [1,6]

The incidence of GISTs is estimated to be 20/1.000.000 persons per year. The median age of the manifestation ranges between 55 and 65 years of age. These tumors are very rare in individuals before the age of 40 and some studies show a small male predominance [1,2,6].

In general the GISTs have a wide clinical manifestation that depends upon the location and the size of the tumor. In colorectal area the tumor may be manifested with lower GI bleeding, perforation, obstruction and pain. The stomach is the most common site of GIST benign tumors, whereas most esophageal and colonic GISTs are malignant [1]. Approximately 20 to 25% of gastric and 40%–50% of small intestinal GISTs, are clinically malignant [9]. GISTs usually metastasize to the liver and lung but in recent publications there have been reports to the skin and brain as sites of metastases [6]. Other case reports described involvement of the omentum, the mesentery and the retroperitoneum, secondary to their GI tract original location [3,9]. Metastases to the bone and soft tissue are very rare. Miettinen et al. reported humeral metastasis and paraspinal soft tissue involvement in two patients with colonic GIST [2].

In the case presented herein, the tumor was misdiagnosed and remained untreated for about 4 years. Our hypothesis is that the tumor could have had escaped complete resection from gynecologists either due to small adhesions within the recto-sigmoid area or due to minimal involvement of the gut wall. Histologically a GIST tumor is very similar to leiomyosarcoma (LMS) but in our case non c-KIT positivity was not checked and the tumor was originally diagnosed as uterine leiomyosarcoma. The patient was treated with chemotherapy and radiotherapy but the GISTs are refractory to both [1]. Therefore, we present the natural history of an EGIST localized in pelvis with pulmonary and soft tissue metastasis and a late manifestation from the area of the resected primary.

The differential diagnosis between leiomysarcoma and GIST remains a challenge. Of a total of 133 rectal and anal GISTs identified in the Armed Forces Institute of Pathology at Washington and in the Haartman Institute of the university of Helsinki, 80 tumors (60%) had been originally diagnosed from other centers as leiomyosarcoma (LMSs), 29 tumors (21.8%) as smooth muscle tumors of uncertain malignant potential, 21 tumors (15.8%) as leiomyoma (LMs) and only 3 tumors (2.25%) as GISTs [8].

Immunohistochemically, the majority of GI mesechymal tumors are GISTs and are strongly c-KIT positive (96–100%) and in particular, esophageal and rectal ones are nearly consistently CD34-positive (95–100%) [1,6].

Our case may be considered as EGIST. Traditionally the EGIST are mesenchymal tumors with similar clinicopathologic and genetic profile localized in the omentum, mesentery and retroperitoneum comprising less than 5% of GISTs [1,2]. In the last few years, few cases have been reported in unusual anatomic location [10,11]. Others suggest that true EGISTs are extremely rare, less than 1,5%, and probably are extramural gastric (omental) or small intestinal (mesenteric) in origin [1]. One of the criteria for diagnosing EGISTs is the recognition of minor association or adhesions with a neighboring gut segment [12]. The prognosis of EGIST, as all GISTs tumors, depends on the mitotic activity, the tumor size, but also the age and location [1,2,5]. Gastric tumors have a less aggressive behavior than intestinal tumors. Size larger than 5 cm is also more malignant and a mitotic count over 50/50 HPF demonstrate a high-grade malignancy. Based on these observations our patient had a very aggressive EGIST with high probability for metastasis.

To the best of our knowledge, this is the first case of EGISTs to metastasize bilaterally in the gluteal region. In a retrospective study of 118 metastasis to soft tissues over a period of 30 year, regardless of the primary, 5 cases were located in the gluteal region, all from carcinomas and melanoma. In only one case located to the abdominal wall, the primary tumor was GIST of the small bowel [13].

Skeletal muscle is resistant to both primary and metastatic cancer. Previous reports have cited various mechanisms as reasons for muscle resistance to malignancy [14-16]. Weiss found that, cancer cells survive best in denervated muscle compared with electrically stimulated muscles [17]. This finding suggests that most cancer cells die soon after haematogenous spread to muscle because of an inhospitable mechanical, pH, and a metabolic environment in normally functioning muscles. Muscles that are injured may have a different mechanical, pH, or metabolic environment that is more favourable to the survival of metastatic cancer cells. We propose that, because of the post-operative radiotherapy, similarly to the muscle injury, the metabolic changes of the gluteal area included in the field of the radiotherapy, probably created a favourable environment for the development of the soft tissue metastasis.

In conclusion, EGISTs are rare tumours of abdominal cavity with potentially high malignancy and metastatic capacity, exhibiting clinical and histological difficulty for a correct diagnosis. Metastatic disease may occur in the soft tissues. Early recognition and prompt diagnosis, will allow the proper treatment to be initiated.

## Consent

Written informed consent was obtained from the patient for publication of this case report and accompanying images. A copy of the written consent is available for review by the Editor-in-Chief of this journal.

## Competing interests

The authors declare that they have no competing interests.

## Authors' contributions

DP, EV and PV initiated and co-wrote the paper and performed the surgical excision of the gluteal metastases. AK analysed the MR images, prepared the illustrations and legend, and performed the proof editing. EG and MT examined the specimen and prepared the histological illustrations.
